# CSF Tau and Amyloid Biomarkers in Cognitive Impairment Across the ALS–FTD Spectrum: A Systematic Narrative Review and Evidence Map

**DOI:** 10.1002/brb3.71740

**Published:** 2026-09-01

**Authors:** Aasim Ali, Muhammad Arif, Usama Ashraf, Saad Elahi, Aswad Mustafa, Mahad Bin Yasir, Waqar Sarfraz, Asad Ullah Ansari, Hafiz Muhammad Yousaf Masood, Muhammad Asad Shabbir, Armaghana Abdullah, Mukesh Kumar Sharma

**Affiliations:** ^1^ Neurology Department Allied Hospital Faisalabad Faisalabad Pakistan; ^2^ Internal Medicine Department Allied Hospital Faisalabad Faisalabad Pakistan; ^3^ Neurology Department Tribhuvan University – Dhankuta Multiple Campus Dhankuta Nepal

**Keywords:** amyloid biomarkers, amyotrophic lateral sclerosis, cognitive impairment, frontotemporal dementia, Tau protein

## Abstract

**Introduction:**

Cognitive impairment is an important non‐motor manifestation of amyotrophic lateral sclerosis (ALS), particularly across the ALS–frontotemporal dementia (ALS–FTD) spectrum. Cerebrospinal fluid (CSF) tau and amyloid biomarkers may reflect nonspecific neurodegenerative injury, concomitant Alzheimer disease (AD) pathology, or distinct cognitive phenotypes. However, available evidence remains limited, fragmented, and methodologically heterogeneous.

**Methods:**

This systematic narrative review and semi‐quantitative evidence map was conducted according to PRISMA 2020 recommendations. PubMed, Scopus, Web of Science, and Google Scholar were searched from database inception through May 2026. Eligible observational studies reported cognition‐specific associations between CSF tau, amyloid, or related neurodegenerative biomarkers and cognitive outcomes in ALS‐spectrum populations. Two reviewers independently screened studies, extracted quantitative effect estimates, and assessed risk of bias using the Newcastle–Ottawa Scale. Cross‐study consistency was summarized using an exploratory semi‐quantitative evidence‐coding framework.

**Results:**

Four observational studies comprising 638 ALS‐spectrum participants fulfilled the eligibility criteria. Total tau and p‐tau181 showed the most reproducible associations with cognition. Total tau correlated with ECAS total (*r* = −0.398, *P* < 0.001) and ALS‐specific cognition (*r* = −0.403, *P* < 0.001), while adjusted multicenter analyses demonstrated associations between p‐tau181 and ECAS total (*β* = −0.03, *p* = 0.006) and memory performance (*β* = −0.04, *p* = 0.003). Both biomarkers received ++ evidence‐map coding. Amyloid findings were more heterogeneous; lower Aβ42/Aβ40 was associated with poorer memory performance (*β* = 0.20, *p* = 0.044), but continuous amyloid–cognition associations were not consistently replicated across cohorts (± evidence). Broader CSF protein‐ratio findings remained exploratory. Clinical, cognitive, assay, and analytical heterogeneity precluded meta‐analysis.

**Conclusions:**

The available evidence, although limited and heterogeneous, suggests that total tau may primarily reflect broader neurodegenerative injury, whereas p‐tau181 and Aβ42/Aβ40 may be more informative for selected cognitive phenotypes or possible AD co‐pathology. These biomarkers should remain research tools until larger, standardized, longitudinal multicenter studies establish their pathological specificity, predictive value, and clinical utility.

## Graphical abstract

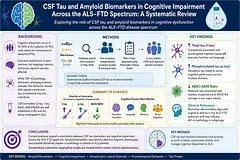

## 1 Introduction

Amyotrophic lateral sclerosis (ALS) is a progressive neurodegenerative disorder characterized by degeneration of upper and lower motor neurons, leading to weakness, respiratory failure, and death (Hardiman et al. 2017). Historically regarded as a pure motor neuron disease, ALS is now recognized as a multisystem neurodegenerative disorder with substantial cognitive and behavioral involvement (Strong et al. 2017). Cognitive and behavioral impairment may occur across a continuum ranging from mild executive dysfunction to ALS–frontotemporal dementia (ALS–FTD), supporting the concept of an ALS–FTD spectrum disorder (Strong et al. 2017; Burrell et al. 2016).

Cognitive dysfunction in ALS is clinically important because it is associated with poorer survival, impaired functional autonomy, reduced decision‐making capacity, and increased caregiver burden (Crockford et al. 2018). Executive dysfunction, verbal fluency impairment, and language abnormalities are among the most frequently recognized ALS‐specific cognitive deficits, while memory and visuospatial impairment may also occur in selected patients (Abrahams et al. 2014). The Edinburgh Cognitive and Behavioural ALS Screen (ECAS) and ALS Cognitive Behavioral Screen (ALS–CBS) were developed to improve detection of cognitive and behavioral changes in ALS while reducing confounding from motor disability (Abrahams et al. 2014; Woolley et al. 2010).

Neuropathologically, the ALS–FTD spectrum is primarily associated with transactive response DNA‐binding protein 43 (TDP‐43) pathology, which appears to spread in a staged pattern across motor and extra‐motor regions (Brettschneider et al. 2013). Accordingly, amyloid‐β and tau abnormalities should not be regarded as defining components of ALS pathophysiology in the same manner as they are in Alzheimer disease (AD). Their investigation in ALS instead addresses a different question: whether AD‐associated biomarker abnormalities identify concomitant AD pathology, reflect nonspecific neuronal injury accompanying ALS, or contribute to the biological phenotyping of cognitively heterogeneous ALS populations. AD‐related biomarker frameworks emphasize amyloid‐beta and tau abnormalities as key biological markers of Alzheimer pathology (Olsson et al. 2016; Jack et al. 2018). Within this framework, these biomarkers have different biological interpretations. Reduced cerebrospinal fluid (CSF) Aβ42 or, more specifically, a reduced Aβ42/Aβ40 ratio is primarily interpreted as evidence of cerebral amyloid dysregulation/deposition, whereas p‐tau181 is more closely related to AD‐type tau pathology. In contrast, total tau is a comparatively nonspecific marker of neuronal or axonal injury and may increase in neurodegenerative conditions without primary tau pathology (Olsson et al. 2016; Jack et al. 2018). Therefore, abnormalities in total tau should not, in isolation, be interpreted as evidence of AD co‐pathology in ALS. CSF biomarkers such as total tau, p‐tau181, Aβ42, Aβ40, and the Aβ42/Aβ40 ratio are therefore increasingly being investigated in ALS cohorts to determine whether they reflect AD co‐pathology, broader neurodegenerative injury, or distinct ALS cognitive phenotypes (Olsson et al. 2016; Jack et al. 2018).

This distinction is particularly important because the cognitive phenotype itself may provide clues to the biological significance of these biomarkers. ALS‐related frontotemporal dysfunction characteristically involves executive, language, and verbal‐fluency domains, whereas disproportionate memory impairment may raise the possibility of additional non‐ALS pathology. Thus, an amyloid‐positive profile, particularly when accompanied by p‐tau181 abnormalities and memory‐predominant impairment, may be more suggestive of concomitant AD‐type biology than an isolated elevation in total tau. Conversely, associations between total tau and broad ECAS impairment may reflect the overall burden of neuronal injury rather than a specific AD process. These interpretations remain hypotheses rather than established diagnostic rules and require evaluation within the clinical and cognitive context of ALS.

The concept of mixed neurodegeneration has therefore become increasingly relevant in ALS research. Overlap between ALS, FTD, and AD‐related pathological processes may be particularly important in cognitively impaired ALS subgroups. APOE‐E4 carriage, amyloid dysregulation, and tau abnormalities may identify biologically distinct ALS phenotypes with increased vulnerability to cognitive decline or memory‐predominant impairment (Maranzano et al. 2024). Importantly, such findings do not imply that AD pathology is the principal cause of cognitive impairment across the ALS–FTD spectrum. Rather, they raise the possibility that AD co‐pathology contributes to cognitive impairment in a subset of patients, while tau abnormalities in others may reflect TDP‐43‐associated or otherwise nonspecific neurodegenerative injury. A third possibility is that combinations of CSF biomarkers identify biological subtypes of ALS with differing cognitive vulnerability even in the absence of pathologically confirmed AD. Understanding these mechanisms is important because cognitive impairment influences prognosis, caregiver burden, clinical decision‐making, and future therapeutic stratification (Crockford et al. 2018).

Despite increasing interest, the literature remains heterogeneous. Studies differ substantially in assay methodology, cognitive subgroup definitions, cognitive assessment tools, and biomarker reporting. They also differ in the biological questions addressed: some examine conventional AD‐related amyloid and tau biomarkers, whereas others evaluate broader neurodegenerative or proteomic signatures associated with cognition. Many investigations focus on diagnostic discrimination between ALS and controls, survival, or disease progression rather than cognition‐domain‐specific biomarker associations. In addition, several studies report correlation coefficients or regression analyses without extractable subgroup means and standard deviations, limiting quantitative synthesis. These differences complicate direct comparison between studies and, importantly, limit the extent to which an observed biomarker–cognition association can be attributed specifically to AD co‐pathology rather than to general neurodegeneration or ALS‐related biological heterogeneity.

Therefore, this systematic narrative review and evidence map aimed to evaluate current evidence regarding CSF tau and amyloid biomarkers and their association with cognitive impairment across the ALS–FTD spectrum. The primary objective was to determine whether the available evidence can distinguish biomarker patterns suggestive of co‐occurring AD pathology from those more plausibly reflecting broader neurodegenerative injury, while also assessing whether these biomarkers may help phenotype cognitive heterogeneity within ALS. Accordingly, we considered total tau principally as a marker of nonspecific neuronal injury, while interpreting p‐tau181 and the Aβ42/Aβ40 ratio more specifically in the context of possible AD co‐pathology, without assuming that abnormal values establish AD as the mechanism of cognitive impairment. The review specifically sought to identify biomarker‐domain patterns, clarify whether tau and amyloid markers appear related to cognitive phenotype or possible AD co‐pathology, and define methodological gaps that currently limit meta‐analysis and clinical translation.

## 2 Methods

### 2.1 Study Design and Reporting

This systematic review was conducted according to the Preferred Reporting Items for Systematic Reviews and Meta‐Analyses (PRISMA) 2020 statement (Page et al. 2021). Because quantitative pooling was not feasible, the review was designed as a systematic narrative review with structured evidence mapping. The synthesis was organized around three biological interpretations: biomarkers potentially reflecting AD co‐pathology, biomarkers representing broader or nonspecific neurodegenerative injury, and biomarker patterns potentially useful for phenotyping cognitive heterogeneity across the ALS–FTD spectrum.

### 2.2 Eligibility Criteria

Eligible studies included observational cohort, cross‐sectional, and case‐control studies evaluating CSF tau, amyloid, or closely related neurodegenerative biomarkers in ALS or ALS–FTD‐spectrum populations. For inclusion in the primary synthesis, studies were required to report cognition‐specific associations between CSF biomarkers and cognitive outcomes. These included ECAS total score, ECAS ALS‐specific or ALS‐nonspecific subscores, executive function, language, verbal fluency, memory, visuospatial function, or formally defined ALS cognitive/behavioral phenotypes.

Biomarkers of interest included total tau, p‐tau181, Aβ42, Aβ40, Aβ42/Aβ40 ratio, Aβ42/p‐tau181 ratio, and broader CSF proteomic markers when directly linked to cognitive outcomes. Terminology was standardized to p‐tau181 when this specific phospho‐tau species was measured.

Studies reporting only diagnostic discrimination between ALS and controls, survival, disease progression, ALS‐versus‐control biomarker differences, or biomarker abnormalities without cognition‐domain‐specific analyses were excluded from the primary synthesis. Reviews, editorials, conference abstracts, animal studies, case reports, and retracted publications were excluded. Studies evaluating relevant biomarkers but not meeting primary eligibility criteria were summarized separately in the supplementary full‐text exclusion table.

### 2.3 Information Sources and Search Strategy

A comprehensive literature search was performed in PubMed, Scopus, Web of Science, and Google Scholar from database inception through May 2026. Search terms included combinations of “amyotrophic lateral sclerosis,” “ALS,” “frontotemporal dementia,” “cognitive impairment,” “ECAS,” “CSF,” “tau,” “phosphorylated tau,” “amyloid‐beta,” “Aβ42,” and “biomarkers.” Boolean operators and database‐specific filters were applied where appropriate to maximize sensitivity. The complete search strategy is presented in Supporting Material 1. Reference lists of eligible studies and relevant reviews were also manually screened.

### 2.4 Study Selection and Data Extraction

Two reviewers independently screened titles and abstracts for eligibility. Full texts of potentially relevant articles were subsequently reviewed independently using predefined inclusion and exclusion criteria. Disagreements were resolved through discussion and consensus.

Data extraction was performed using standardized forms and included study design, country, sample size, demographic characteristics, cognitive assessment methods, cognitive domains evaluated, CSF biomarkers, assay platforms, principal cognition‐related findings, statistical methods, and availability of quantitative data. Where available, effect estimates were extracted, including correlation coefficients, regression coefficients, odds ratios, 95% confidence intervals, and *P* values. Adjusted estimates were preferentially recorded, together with the covariates included in the corresponding model. Unadjusted correlation analyses were retained but explicitly distinguished from multivariable‐adjusted results. Relevant potential confounders, including age, disease duration, ALS severity, APOE genotype, and genetic characteristics, were recorded when reported.

### 2.5 Risk‐of‐Bias Assessment

Methodological quality was assessed using the Newcastle–Ottawa Scale for observational studies, with adaptation where required for cross‐sectional designs. Domains included participant selection, comparability of study groups, and outcome assessment. Particular attention was given to cognitive subgroup definitions, biomarker assay methods, confounder adjustment, and statistical reporting. The risk‐of‐bias assessment is presented in Figure S1.

Risk‐of‐bias findings were incorporated into interpretation rather than considered separately. Greater weight was given to findings from prospective or multicenter cohorts, standardized cognitive assessments, adequately characterized populations, and analyses adjusted for relevant confounders. Findings from retrospective cohorts, small biomarker subsets, or predominantly unadjusted correlation analyses were interpreted more cautiously.

### 2.6 Evidence Mapping and Data Synthesis

A qualitative narrative synthesis with semi‐quantitative structured evidence mapping was performed. Meta‐analysis was considered but was not undertaken because of substantial clinical, methodological, and analytical heterogeneity. Major sources included differences in cognitive subgroup definitions, ECAS thresholds, biomarker assay platforms, availability of subgroup data, statistical approaches, and covariate adjustment.

Heterogeneity was examined across four domains: clinical heterogeneity, including age, ALS phenotype, disease duration, genetic background, and APOE status; cognitive heterogeneity, including differing cognitive classifications and domain‐specific outcomes; biomarker heterogeneity, including assay platform, analyte definitions, biomarker ratios, and absence of harmonized thresholds; and analytical heterogeneity, including correlation versus regression approaches and differences in confounder adjustment. These factors were considered when determining whether apparently similar findings represented biological consistency or could reflect methodological variation or confounding.

Several studies reported correlations or regression estimates without extractable subgroup means and standard deviations, while others evaluated broader proteomic signatures rather than conventional tau or amyloid biomarkers. Pooling these heterogeneous data could therefore have produced misleading summary estimates.

For each biomarker–cognitive‐domain association, we summarized the number of contributing studies, direction of effect, statistical significance, available effect estimates, methodological quality, and presence of conflicting findings. Evidence consistency was coded using an exploratory scale: “+++” indicated a replicated, directionally consistent association across at least two studies with supportive evidence from a methodologically stronger study and no major conflicting evidence; “++” indicated concordant findings across at least two studies but with important methodological limitations; “+” indicated a signal supported by a single study; “±” indicated inconsistent or conflicting findings; and “0” indicated no demonstrated significant association. This coding system was used solely as a transparent evidence‐mapping aid and was not intended as a validated certainty‐of‐evidence framework.

Studies were synthesized according to biomarker category, cognitive domain, direction and magnitude of association, consistency across studies, and methodological robustness. Conventional tau and amyloid biomarkers were considered separately from broader exploratory proteomic markers to avoid implying equivalent biological specificity.

## 3 Results

### 3.1 Study Selection and Characteristics

**FIGURE 1:**
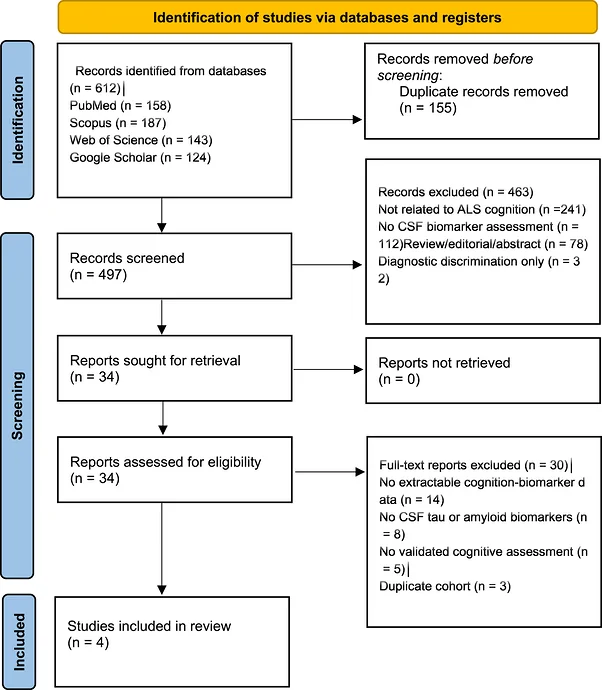
PRISMA flow diagram. Study identification, screening, eligibility, and inclusion process.

Database searches identified 612 records from PubMed, Scopus, Web of Science, and Google Scholar. After removal of 115 duplicates, 497 records underwent title and abstract screening, of which 463 were excluded. Thirty‐four full‐text articles were assessed for eligibility, and four studies fulfilled the final inclusion criteria for qualitative synthesis and structured evidence mapping. The study selection process is shown in Figure 1. Studies evaluating tau, amyloid, or related neurodegenerative biomarkers without cognition‐domain‐specific CSF biomarker associations were excluded from the primary synthesis and are summarized in Table S1.

The four included studies were published between 2024 and 2026 and comprised observational cohorts from Germany, Italy, and Sweden (Kasper et al. 2026; Wölfel et al. 2025; Maranzano et al. 2024; Öijerstedt et al. 2026). ECAS was the principal cognitive assessment across all studies. Three studies directly evaluated conventional CSF amyloid and tau biomarkers (Kasper et al. 2026; Wölfel et al. 2025; Maranzano et al. 2024), whereas Öijerstedt et al. (2026) investigated a broader panel of neurodegeneration‐related CSF proteins and protein ratios. Considerable variation was present in cohort composition, biomarker availability, assay methodology, cognitive classification, statistical analysis, and confounder adjustment. These characteristics are summarized in Table 1.

**TABLE 1:** Characteristics and methodological features of included studies.

Study	Design and sample	Cognitive assessment	CSF biomarkers/platform	Main methodological considerations
Kasper et al. (2026)	Germany; prospective multicenter cohort; 192 ALS/ALS–FTD participants and 100 healthy controls; smaller subsets contributed to CSF–cognition analyses	ECAS with age‐ and education‐standardized scores; ALS cognitive phenotyping	Aβ38, Aβ40, Aβ42, total tau, p‐tau181, Aβ42/Aβ40, Aβ42/p‐tau181; automated CSF assays	Strongest design among included studies, with multicenter recruitment and adjusted analyses; however, biomarker availability resulted in smaller analytic subsets and the principal CSF–cognition analyses were predominantly cross‐sectional
Maranzano et al. (2024)	Italy; observational cohort; 281 ALS patients, with CSF biomarker data available for 105	ECAS total and domain‐specific scores	Aβ42, Aβ40, Aβ42/Aβ40, total tau, p‐tau181; Lumipulse chemiluminescence enzyme immunoassay	CSF biomarkers were available in only 105/281 participants; predominantly cross‐sectional analyses and differing regression models limited direct comparability with other studies
Wölfel et al. (2025)	Germany; retrospective observational cohort; 99 ALS patients, 73 with CSF biomarker measurements	ECAS total, ALS‐specific, and ALS‐nonspecific scores	Total tau, p‐tau181, Aβ42, Aβ40; routine clinical CSF assays	Retrospective design, relatively small CSF subset, and predominantly unadjusted correlation analyses increased susceptibility to residual confounding
Öijerstedt et al. (2026)	Sweden; observational cohort; 66 ALS patients; 44% had ECAS‐defined cognitive impairment	ECAS; cognitive impairment defined as total score <108	47 neurodegeneration‐related CSF proteins and protein ratios; Luminex/FlexMap 3D multiplex platform	Exploratory proteomic design rather than a conventional amyloid/tau study; small sample, large number of candidate ratios, and lack of external validation limit generalizability

**Abbreviations**: ALS, amyotrophic lateral sclerosis; ALSci, ALS with cognitive impairment; ALS–FTD, ALS with frontotemporal dementia; Aβ, amyloid‐β; CLEIA, chemiluminescence enzyme immunoassay; ECAS, Edinburgh Cognitive and Behavioural ALS Screen; p‐tau181, tau phosphorylated at threonine 181.

### 3.2 Biomarker–Cognition Patterns

Across the conventional biomarker studies, tau‐related measures demonstrated the most reproducible associations with cognitive performance, but the evidence was not completely uniform. The most consistent signal involved total tau and p‐tau181, with replication across two independent cohorts and stronger support for p‐tau181 from adjusted multicenter analyses (Kasper et al. 2026; Wölfel et al. 2025). However, tau associations reported in Maranzano et al. (2024) were attenuated after adjustment for Aβ40, indicating that tau‐related findings should not be interpreted as universally independent across cohorts.

Amyloid‐related findings were more heterogeneous. The Aβ42/Aβ40 ratio showed its clearest relationship with memory‐predominant cognitive impairment and APOE‐ε4‐associated phenotypes rather than with broad ECAS impairment (Kasper et al. 2026; Maranzano et al. 2024). Individual Aβ42 and Aβ40 concentrations showed associations with several cognitive domains in one cohort but no significant associations in another (Wölfel et al. 2025; Maranzano et al. 2024). This inconsistency reduces confidence in individual amyloid species as general markers of ALS‐related cognitive dysfunction and suggests that amyloid‐ratio abnormalities may be more informative when interpreted in the context of possible AD co‐pathology.

The proteomic findings from Öijerstedt et al. (2026) provide complementary evidence that combinations or ratios of CSF proteins may capture cognitive heterogeneity in ALS. However, because this study did not primarily evaluate conventional AD biomarkers, its findings were considered supportive evidence for broader biological phenotyping rather than direct confirmation of amyloid‐ or tau‐related associations.

Cross‐study consistency, representative quantitative estimates, methodological robustness, and conflicting findings were therefore integrated into the structured evidence map presented in Table 2.

**TABLE 2:** Semi‐quantitative evidence map of CSF biomarker associations with cognitive impairment across the ALS–FTD spectrum.

Biomarker pattern	Studies contributing	Main cognitive phenotype/domain	Representative quantitative evidence	Cross‐study interpretation	Evidence code
**Total tau**	Kasper et al. (2026); Maranzano et al. (2024); Wölfel et al. (2025)	ECAS total, ALS‐specific, and ALS‐nonspecific cognition	Wölfel: ECAS total *r* = −0.398, *P* < 0.001; ALS‐specific *r* = −0.403, *P* < 0.001; ALS‐nonspecific *r* = −0.288, *p* = 0.013. Kasper: significant cognitive‐group differences, *p* = 0.002	Repeated association in two cohorts, but not uniformly independent after adjustment; biologically compatible with broader neuronal/neurodegenerative injury rather than an AD‐specific signal	**++**
**p‐tau181**	Kasper et al. (2026); Maranzano et al. (2024); Wölfel et al. (2025)	Global cognition, language, memory, ALS‐specific and ALS‐nonspecific cognition	Kasper: ECAS total *β* = −0.03, *p* = 0.006; memory *β* = −0.04, *p* = 0.003; language *β* = −0.02, *p* = 0.004. Wölfel: ECAS total *r* = −0.242, *p* = 0.039; ALS‐specific *r* = −0.241, *p* = 0.040	Directionally replicated association supported by adjusted multicenter evidence, although not consistently independent across all cohorts	**++**
**Aβ42/Aβ40 ratio**	Kasper et al. (2026); Maranzano et al. (2024)	Memory impairment, cognitive subgroup status, APOE‐ε4‐related phenotype	Maranzano: memory *β* = 0.20, 95% CI 0.003–0.39; *p* = 0.044. Kasper: lower ratios in ALSci and ALS–FTD than controls, but no significant continuous ECAS association	Predominantly subgroup‐ and memory‐related signal; potentially more consistent with AD‐type co‐pathology than generalized ALS cognitive impairment	±
**Aβ42/p‐tau181 ratio**	Kasper et al. (2026)	Cognitive subgroup status	Lower values reported in ALSci and ALS–FTD than controls	Single‐study signal compatible with possible AD‐related co‐pathology; requires replication	**+**
**Aβ42**	Maranzano et al. (2024); Wölfel et al. (2025)	ECAS total, memory, fluency, and other cognitive domains	Maranzano: ECAS total *β* = 0.27, 95% CI 0.10–0.43; *p* = 0.001. Wölfel: ECAS total *r* = −0.098, *p* = 0.409	Conflicting findings across cohorts	±
**Aβ40**	Maranzano et al. (2024); Wölfel et al. (2025)	ECAS total, ALS‐specific cognition, fluency	Maranzano: ECAS total *β* = 0.26, *p* = 0.005; fluency *β* = 0.36, *P* < 0.001. Wölfel: ECAS total *r* = −0.191, *p* = 0.105	Conflicting findings across cohorts	±
**Broader CSF protein ratios**	Öijerstedt et al. (2026)	ECAS total and ECAS‐defined cognitive impairment	PTPRN2/GAP43: *β* = 7.48, 95% CI 3.66–11.29; cross‐validated *R* ^2^ = 0.34; AUC 0.89, 95% CI 0.80–0.97	Promising exploratory single‐study evidence for broader cognitive phenotyping; not direct evidence for conventional amyloid/tau biomarkers	**+**

*Note*: Evidence coding: +++ = replicated and directionally consistent evidence across ≥2 studies, including a methodologically stronger study, without major conflicting evidence; ++ = concordant evidence across ≥2 studies but with important methodological limitations or some inconsistency; + = signal from a single study; ± = inconsistent or conflicting findings; 0 = no demonstrated significant association. *These categories are descriptive evidence‐mapping labels and should not be interpreted as a validated certainty‐of‐evidence grading system*.

### 3.3 Methodological Heterogeneity and Risk of Bias

Substantial heterogeneity was identified across four interrelated domains. Clinical heterogeneity included differences in age distribution, ALS phenotype, disease duration, APOE status, and biomarker availability. Cognitive heterogeneity arose from differences in ECAS classification, domain definitions, and approaches to defining cognitive impairment. Biomarker heterogeneity reflected differences in analytes, assay platforms, biomarker ratios, and absence of harmonized thresholds, while analytical heterogeneity included unadjusted correlations, multivariable regression, penalized modeling, and variable adjustment for potential confounders. These differences prevented calculation of a meaningful common effect estimate and supported the decision not to perform meta‐analysis.

Risk of bias was overall moderate but was incorporated directly into interpretation of the evidence rather than treated as an isolated quality score. Findings derived from prospective or multicenter cohorts, standardized cognitive assessments, and adjusted statistical models were given greater interpretative weight, whereas retrospective analyses, small biomarker subsets, and predominantly unadjusted correlations were considered less robust. Consequently, evidence‐map classifications reflected both cross‐study consistency and methodological quality rather than simply the number of statistically significant findings.

**FIGURE 2:**
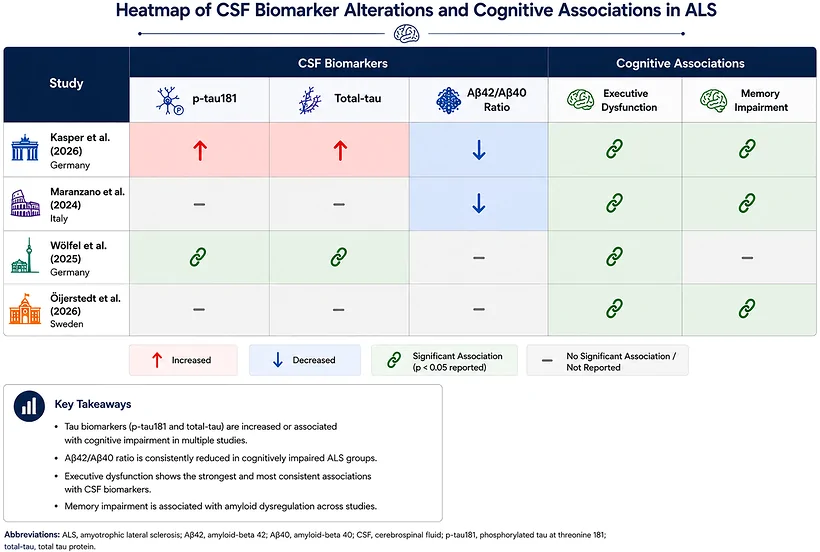
CSF biomarker–cognition heatmap in ALS. Qualitative summary of CSF biomarker alterations and cognitive associations across included studies. Tau‐related markers showed more consistent associations, whereas amyloid findings were heterogeneous. This figure represents qualitative patterns from four observational studies and does not show effect sizes, causal relationships, or formal certainty of evidence.

Figure 2 summarizes the principal biomarker–cognition patterns identified in the evidence map. The figure represents qualitative cross‐study patterns and should not be interpreted as displaying comparative effect sizes, causal relationships, or formal certainty of evidence.

## 4 Discussion

The present systematic narrative review and evidence map summarizes emerging evidence regarding CSF tau and amyloid biomarkers in cognitive impairment across the ALS–FTD spectrum. Because only four eligible observational studies were identified and heterogeneity was substantial, the principal contribution is a structured interpretation of biomarker–cognition patterns rather than a pooled estimate. The evidence suggests different biological meanings for individual biomarkers. Total tau appears more consistent with nonspecific neuronal or axonal injury, whereas p‐tau181 and the Aβ42/Aβ40 ratio may be more informative for possible concomitant AD pathology. These interpretations are not mutually exclusive and must remain anchored to TDP‐43 as the principal proteinopathy underlying the ALS–FTD spectrum (Brettschneider et al. 2013).

Among the biomarkers studied, tau‐related measures showed the most reproducible associations with cognitive performance, although the evidence was neither uniform nor sufficient to establish causality. Kasper et al. (2026) demonstrated associations between CSF tau biomarkers and poorer ECAS performance across several domains, while Wölfel et al. (2025) independently reported negative relationships between total tau, p‐tau181, and ECAS scores. However, confidence differs by study design. Kasper et al. (2026) provide stronger evidence because it was derived from a prospective multicenter cohort, used standardized cognitive phenotyping, and incorporated multivariable analyses. Wölfel et al. (2025) was retrospective, included a smaller CSF subset, and relied mainly on unadjusted correlations, making residual confounding more likely. Maranzano et al. (2024) add caution because initial associations of total tau and p‐tau181 with verbal fluency were attenuated after adjustment for Aβ40.

The biological interpretation of total tau requires particular caution. ALS is primarily characterized by TDP‐43 pathology rather than a primary tauopathy (Brettschneider et al. 2013). Elevated CSF total tau can occur in several neurological disorders as a consequence of neuronal injury and axonal degeneration and therefore cannot, in isolation, be regarded as evidence of AD co‐pathology. Its association with broad ECAS impairment may instead reflect the overall burden or anatomical extent of neurodegeneration. This interpretation is consistent with previous ALS biomarker studies in which CSF tau was evaluated as a diagnostic or prognostic marker independent of clinically evident AD (Grossman et al. 2014; Scarafino et al. 2018; Wilke et al. 2015). Accordingly, total tau may be best considered a marker of neurodegenerative intensity rather than a disease‐specific signal within the ALS–FTD spectrum.

p‐tau181 warrants a different, although still cautious, interpretation. Within AD biomarker frameworks, phosphorylated tau is more closely linked to AD‐type tau pathology than total tau (Olsson et al. 2016; Jack et al. 2018). Associations between p‐tau181 and global and domain‐specific cognitive performance in ALS therefore raise the possibility that AD‐related processes contribute to cognitive impairment in a subset of patients. Nevertheless, p‐tau181 abnormalities in ALS should not automatically be equated with clinically meaningful AD co‐pathology. The findings of Kasper et al. (2026) are particularly informative because biomarker associations coexisted with neuropathological evidence implicating regional TDP‐43 pathology in cognitive dysfunction. This supports a model in which AD‐related biology may modify the cognitive phenotype in some individuals while TDP‐43‐associated neurodegeneration remains central to ALS‐related cognitive impairment.

Amyloid‐related findings were less consistent across studies. The clearest signal involved the Aβ42/Aβ40 ratio and memory‐predominant impairment rather than generalized executive or language dysfunction. Maranzano et al. (2024) identified lower Aβ42/Aβ40 ratios in association with poorer memory performance and APOE‐ε4 carriage. Kasper et al. (2026) also observed lower amyloid‐related ratios in cognitively impaired ALS and ALS–FTD subgroups, although continuous amyloid measures did not independently predict ECAS performance in adjusted analyses. This distinction is important because an abnormal amyloid profile may define a biological subgroup without necessarily showing a linear relationship with overall cognitive severity.

A profile combining reduced Aβ42/Aβ40, abnormal p‐tau181, older age, APOE‐ε4 carriage, and disproportionate memory impairment would therefore be more compatible with possible concomitant AD biology than an isolated increase in total tau (Olsson et al. 2016; Jack et al. 2018; Maranzano et al. 2024). Even such a profile should remain suggestive rather than diagnostic, because the included studies did not systematically confirm AD pathology using amyloid or tau imaging or neuropathology in all participants. Individual Aβ42 and Aβ40 concentrations were also inconsistent across cohorts: Maranzano et al. (2024) reported associations with several ECAS domains, whereas Wölfel et al. (2025) found no significant cognitive relationships. These conflicting findings argue against the use of single amyloid species as general markers of ALS‐related cognitive impairment.

The available data therefore argue against directly transferring the AD A/T model into ALS. Amyloid and tau biomarkers may identify patients with concomitant AD‐type biology or help distinguish them from those whose cognitive dysfunction reflects frontotemporal network degeneration or broader neuronal injury. These categories are not yet established as biologically discrete, and mixed mechanisms are likely.

The study by Öijerstedt et al. (2026) broadens this concept by suggesting that combinations or ratios of CSF proteins may capture cognitive heterogeneity more effectively than single analytes. However, its exploratory proteomic design, small sample size, and lack of external validation mean that it should not be treated as direct confirmation of conventional tau or amyloid findings. Rather, it supports the hypothesis that multidimensional biomarker profiles may ultimately be more informative than isolated markers for characterizing cognitive phenotypes in ALS.

These findings should also be considered within the wider ALS biomarker landscape. Neurofilament‐based measures are established markers of neuroaxonal injury, disease activity, and prognosis, whereas CSF tau and amyloid biomarkers are not routine tests for ALS cognition. Their potential value is therefore complementary. Neurofilaments may reflect neuroaxonal degeneration, while tau and amyloid profiles may add information about cognitive phenotype or accompanying neurodegenerative pathology. Whether these biomarkers provide clinically meaningful information beyond neurofilaments, genetics, imaging, and standardized cognitive assessment remains unknown.

### 4.1 Methodological Heterogeneity and Critical Appraisal

Methodological heterogeneity is central to interpreting the inconsistencies across studies. First, CSF measurement methods differed substantially, ranging from automated amyloid/tau immunoassays to routine clinical assays and multiplex antibody‐based proteomic platforms (Kasper et al. 2026; Wölfel et al. 2025; Maranzano et al. 2024; Öijerstedt et al. 2026). Differences in analytical sensitivity, calibration, preanalytical handling, laboratory reference ranges, and biomarker thresholds may produce systematic variation in measured concentrations. Consequently, absolute values cannot be assumed to be directly comparable across cohorts.

Second, cognitive classification was not standardized. Although ECAS was used across the included studies, investigators differed in thresholds for impairment, use of age‐ and education‐adjusted norms, subdivision into ALS‐specific and ALS‐nonspecific domains, and classification of ALS cognitive phenotypes. Such differences can alter the proportion of participants classified as impaired and may dilute or exaggerate biomarker associations. This is especially relevant for memory, which may signal a different biological process from the executive, language, and fluency deficits more typically emphasized in ALS.

Third, analytical approaches varied considerably. Wölfel et al. (2025) predominantly reported unadjusted correlations, whereas Kasper et al. (2026) used multivariable models and Maranzano et al. (2024) incorporated relevant covariates. Failure to adjust for age may confound associations because both cognition and AD‐related biomarkers vary with age. Similarly, lack of adjustment for APOE genotype could overestimate amyloid–cognition relationships if APOE‐ε4 influences both amyloid biology and cognitive vulnerability. Disease duration, ALS severity, education, nutritional status, and genetic background represent additional potential confounders.

Fourth, cohort composition differed geographically and clinically. Participants were recruited in Germany, Italy, and Sweden and may differ in referral pathways, demographics, genetics, ALS phenotypes, and APOE distribution (Kasper et al. 2026; Wölfel et al. 2025; Maranzano et al. 2024; Öijerstedt et al. 2026). Several studies also had smaller biomarker subsets than their parent cohorts, creating the possibility of selection bias if individuals who underwent lumbar puncture differed systematically from those without CSF data. Most biomarker–cognition relationships were cross‐sectional, meaning that temporal direction cannot be determined. Biomarker abnormalities may precede cognitive decline, arise as a consequence of more extensive neurodegeneration, or simply coexist with cognitive impairment.

These limitations explain why quantitative pooling was inappropriate. More importantly, heterogeneity identifies the methodological features that future studies must standardize. The evidence base should therefore be interpreted as an emerging hierarchy rather than four equally weighted studies. Kasper et al. (2026) offer the strongest overall evidence because of its multicenter design, adjusted analyses, and integration of biomarker and neuropathological information, although the relevant CSF–cognition analyses remained largely cross‐sectional. Maranzano et al. (2024) contribute important evidence regarding APOE‐ε4, amyloid biology, and memory phenotype, but only a subset had CSF measurements. Wölfel et al. (2025) provide independent replication of tau–ECAS relationships but is limited by retrospective design and unadjusted analyses. Öijerstedt et al. (2026) extend the framework toward multianalyte phenotyping but remains hypothesis‐generating.

### 4.2 Clinical Implications

Clinically, reliable biomarkers could improve prognostication, patient stratification, and recognition of mixed neurodegenerative pathology. A future application may be distinguishing conventional ALS–FTD‐spectrum dysfunction from memory‐predominant phenotypes in which concomitant AD pathology is considered. These biomarkers should remain research tools because no included study established validated clinical cut‐offs or demonstrated clinical utility.

### 4.3 Implications for Future Studies

Future investigations should prioritize adequately powered, prospective, multicenter longitudinal cohorts with serial cognitive and biomarker measurements. Cognitive phenotyping should follow harmonized ALS–FTD spectrum criteria and use validated, language‐specific ECAS norms adjusted for age and education. Domain‐specific outcomes, including executive function, language, verbal fluency, memory, visuospatial performance, ECAS ALS‐specific and ALS‐nonspecific scores, should be reported alongside global cognitive classifications.

CSF collection, storage, preanalytical processing, assay platforms, and biomarker cut‐offs should be standardized across centers. Amyloid analyses should routinely adjust for age and APOE genotype, while models evaluating ALS‐related cognitive heterogeneity should also consider disease duration, ALSFRS‐R, site of onset, education, nutritional status, and relevant ALS‐associated genetic variants where available. Complete adjusted effect estimates with confidence intervals should be reported to permit future quantitative synthesis.

Longitudinal studies should determine whether baseline or changing biomarker profiles predict subsequent cognitive decline rather than merely correlate with contemporaneous ECAS performance. Tau and amyloid measures should be evaluated alongside neurofilaments, plasma biomarkers, imaging, and genetic data. Where feasible, amyloid‐ and tau‐PET or neuropathological confirmation should distinguish true AD co‐pathology from nonspecific biomarker abnormalities. Integration with TDP‐43 markers will be important for defining the relative contribution of ALS‐specific and AD‐related mechanisms. Promising multianalyte signatures should undergo external validation before claims of clinical utility are made.

### 4.4 Strengths and Limitations

This review has several strengths, including a systematic search strategy, cognition‐specific eligibility criteria, extraction of quantitative effect estimates, risk‐of‐bias assessment, and structured evidence mapping. Separating conventional AD‐associated biomarkers from broader proteomic markers and integrating methodological quality into interpretation reduces the risk of treating biologically different findings as equivalent. Nevertheless, conclusions are constrained by the small number of eligible studies, observational designs, heterogeneous analytical methods, small CSF subgroups, and predominantly cross‐sectional analyses. Publication bias could not be meaningfully assessed, and selective reporting cannot be excluded. The evidence map should therefore be regarded as hypothesis‐generating rather than a basis for clinical implementation.

## 5 Conclusion

The available evidence, although limited and heterogeneous, is consistent with the hypothesis that CSF tau and amyloid biomarkers may reflect distinct aspects of cognitive dysfunction across the ALS–FTD spectrum. Total tau appears more plausibly related to broader neurodegenerative injury, whereas p‐tau181 and the Aβ42/Aβ40 ratio may be more informative for possible concomitant AD‐type biology, particularly in memory‐predominant phenotypes. Current findings do not establish AD co‐pathology as a general explanation for cognitive impairment in ALS, and TDP‐43‐associated neurodegeneration remains central. Larger standardized longitudinal studies are required to establish pathological specificity, predictive validity, incremental value, and clinical utility before these biomarkers enter routine ALS cognitive assessment.

## Author Contributions

**Aasim Ali** conceptualized and designed the study, conducted the literature search, performed data extraction and formal analysis, and drafted the original manuscript. **Muhammad Arif**, **Usama Ashraf**, **Saad Elahi**, **Aswad Mustafa**, **Mahad Bin Yasir**, **Waqar Sarfraz**, **Asad Ullah Ansari**, **Hafiz Muhammad Yousaf Masood**, and **Muhammad Asad Shabbir** contributed to study screening, data collection, interpretation of findings, and critical revision of the manuscript. **Armaghana Abdullah** contributed to supervision, manuscript review, and final approval. **Mukesh Kumar Sharma** supervised the study, provided senior intellectual input, reviewed the manuscript critically, and approved the final version. All authors reviewed and approved the final manuscript.

## Funding

The authors have nothing to report.

## Declaration of Artificial Intelligence Tools

(ChatGPT) was used only for language editing and improving readability. The authors take full responsibility for the content of the manuscript.

## Ethics Statement

Ethical approval was not required because this systematic review used previously published aggregate data and did not involve new human participant recruitment or individual patient‐level data.

## Conflicts of Interest

All authors have no conflict of interest to disclose. The authors declare that they have no known competing financial interests or personal relationships that could have appeared to influence the work reported in this paper.

## Supporting information




**Supplementary Table**: brb371740‐sup‐0001‐TableS1.docx

**Supplementary Material**: brb371740‐sup‐0002‐SuppMat.docx

**Supplementary Figure**: brb371740‐sup‐0003‐FigureS1.pdf

## Data Availability

All data generated during this study is available on request. All data analyzed in this systematic review were derived from published studies and are reported within the article and its supplementary materials. Additional extracted data are available from the corresponding author on reasonable request.

## References

[brb371740-bib-0001] Abrahams, S. , J. Newton , E. Niven , J. Foley , and T. H. Bak . 2014. “Screening for Cognition and Behaviour Changes in ALS.” Amyotrophic Lateral Sclerosis and Frontotemporal Degeneration 15, no. 1‐2: 9–14. 10.3109/21678421.2013.805784. 23781974

[brb371740-bib-0002] Brettschneider, J. , K. Del Tredici , J. B. Toledo , et al. 2013. “Stages of pTDP‐43 Pathology in Amyotrophic Lateral Sclerosis.” Annals of Neurology 74, no. 1: 20–38. 10.1002/ana.23937. 23686809 PMC3785076

[brb371740-bib-0003] Burrell, J. R. , G. M. Halliday , J. J. Kril , et al. 2016. “The Frontotemporal Dementia‐Motor Neuron Disease Continuum.” Lancet 388, no. 10047: 919–931. 10.1016/S0140-6736(16)00737-6. 26987909

[brb371740-bib-0004] Crockford, C. , J. Newton , K. Lonergan , et al. 2018. “ALS‐Specific Cognitive and Behavior Changes Associated With Advancing Disease Stage in ALS.” Neurology 91, no. 15: e1370–e1380. 10.1212/WNL.0000000000006317. 30209236 PMC6177274

[brb371740-bib-0005] Grossman, M. , L. Elman , L. McCluskey , et al. 2014. “Phosphorylated Tau as a Candidate Biomarker for Amyotrophic Lateral sclerosis.” JAMA Neurology 71, no. 4: 442. 10.1001/jamaneurol.2013.6064. 24492862 PMC3989393

[brb371740-bib-0006] Hardiman, O. , A. Al‐Chalabi , A. Chio , et al. 2017. “Amyotrophic Lateral Sclerosis.” Nature Reviews Disease Primers 3: 17071. 10.1038/nrdp.2017.71. 28980624

[brb371740-bib-0007] Jack, C. R. , D. A. Bennett , K. Blennow , et al. 2018. “NIA‐AA Research Framework: Toward a Biological Definition of Alzheimer's Disease.” Alzheimer's & Dementia 14, no. 4: 535–562. 10.1016/j.jalz.2018.02.018. PMC5958625 29653606

[brb371740-bib-0008] Kasper, E. , A. Lehto , A. Jürs , et al. 2026. “Alzheimer's Disease Co‐Pathology and Cognitive Impairment in Amyotrophic Lateral Sclerosis.” Annals of Neurology 100, no. 1: 123–138. 10.1002/ana.78227. 42112660 PMC13327596

[brb371740-bib-0009] Maranzano, A. , F. Verde , A. Dubini , et al. 2024. “Association of APOE Genotype and Cerebrospinal Fluid Aβ and Tau Biomarkers With Cognitive and Motor Phenotype in amyotrophic Lateral sclerosis.” European Journal of Neurology 31, no. 9: e16374. 10.1111/ene.16374. 38853763 PMC11295165

[brb371740-bib-0010] Öijerstedt, L. , S. Mravinacová , J. Olofsson , et al. 2026. “Ratios of CSF Proteins Reflect Cognitive Function in ALS.” Alzheimer's Research & Therapy 18, no. 1: 46. 10.1186/s13195-026-01976-y. PMC12931033 41618346

[brb371740-bib-0011] Olsson, B. , R. Lautner , U. Andreasson , et al. 2016. “CSF and Blood Biomarkers for the Diagnosis of Alzheimer's Disease: A Systematic Review and Meta‐analysis.” Lancet Neurology 15, no. 7: 673–684. 10.1016/S1474-4422(16)00070-3. 27068280

[brb371740-bib-0012] Page, M. J. , J. E. McKenzie , P. M. Bossuyt , et al. 2021. “The PRISMA 2020 Statement: An Updated Guideline for Reporting Systematic Reviews.” BMJ 372: n71. 10.1136/bmj.n71. 33782057 PMC8005924

[brb371740-bib-0013] Scarafino, A. , E. D'Errico , A. Introna , et al. 2018. “Diagnostic and Prognostic Power of CSF Tau in Amyotrophic Lateral Sclerosis.” Journal of Neurology 265, no. 10: 2353–2362. 10.1007/s00415-018-9008-3. 30116940

[brb371740-bib-0014] Strong, M. J. , S. Abrahams , L. H. Goldstein , et al. 2017. “Amyotrophic Lateral Sclerosis‐frontotemporal Spectrum Disorder (ALS‐FTSD): Revised Diagnostic Criteria.” Amyotrophic Lateral Sclerosis and Frontotemporal Degeneration 18, no. 3‐4: 153–174. 10.1080/21678421.2016.1267768. 28054827 PMC7409990

[brb371740-bib-0015] Wilke, C. , C. Deuschle , T. W. Rattay , W. Maetzler , and M. Synofzik . 2015. “Total Tau Is Increased, but Phosphorylated Tau Not Decreased, in Cerebrospinal Fluid in Amyotrophic Lateral sclerosis.” Neurobiology of Aging 36, no. 2: 1072–1074. 10.1016/j.neurobiolaging.2014.10.019. 25453560

[brb371740-bib-0016] Wölfel, S. M. , C. N. Widmann , S. Castro‐Gomez , P. Weydt , P. Tacik , and M. T. Heneka . 2025. “Cognitive Capacity in Amyotrophic Lateral Sclerosis: The Value of Diagnostic Markers in Cerebrospinal Fluid and the Influence of Nutrition and Pulmonary Function.” Brain Communications 7, no. 2: fcaf137. 10.1093/braincomms/fcaf137. 40241787 PMC12001800

[brb371740-bib-0017] Woolley, S. C. , M. K. York , D. H. Moore , et al. 2010. “Detecting Frontotemporal Dysfunction in ALS: Utility of the ALS Cognitive Behavioral Screen (ALS‐CBS™).” Amyotrophic Lateral Sclerosis 11, no. 3: 303–311. 10.3109/17482961003727954. 20433413

