# Supplementary material for: CSF Tau and Amyloid Biomarkers in Cognitive Impairment Across the ALS–FTD Spectrum: A Systematic Narrative Review and Evidence Map
Source: Brain Behav. 2026 Sep 1;16(9):e71740. doi: 10.1002/brb3.71740 (PMC13531366; doi:10.1002/brb3.71740)
Supplement: Supplementary file 1 [file BRB3-16-e71740-s002.pdf]

Supplementary Figure S1. Risk-of-bias assessment of included studies

Adapted Newcastle-Ottawa assessment focused on CSF biomarker associations with ALS cognition

Study	Participant selection	Cognitive assessment	CSF biomarker measurement	Confounding / comparability	Reporting / extractability	Overall judgment
Kasper et al. 2026	L	L	L	S	S	S
Maranzano et al. 2024	L	L	L	S	S	S
Wölfel et al. 2025	S	L	L	S	S	S
Öijerstedt et al. 2026	S	L	S	S	H	S

L = Low risk; S = Some concerns; H = High risk

Abbreviations: ALS, amyotrophic lateral sclerosis; CSF, cerebrospinal fluid.
Judgments reflect risk of bias for the review outcome: association between CSF biomarkers and cognition-specific ALS outcomes.
