# Supplementary material for: CSF Tau and Amyloid Biomarkers in Cognitive Impairment Across the ALS–FTD Spectrum: A Systematic Narrative Review and Evidence Map
Source: Brain Behav. 2026 Sep 1;16(9):e71740. doi: 10.1002/brb3.71740 (PMC13531366; doi:10.1002/brb3.71740)
Supplement: Supplementary file 2 [file BRB3-16-e71740-s001.docx]

### Supplementary Table S1. Full-text articles excluded from the primary synthesis and reasons for exclusion

| **No.** | **Excluded full-text study / record** | **Biomarker or topic evaluated** | **Reason for exclusion from primary synthesis** | **Use in revised manuscript** |
| --- | --- | --- | --- | --- |
| 1 | Grossman et al., 2014 | CSF p-tau181, total tau, p-tau:t-tau ratio | CSF tau study in ALS, but cognition was limited mainly to global MMSE and disease-severity measures; no ALS cognition-domain-specific CSF biomarker analysis. | Supportive background only |
| 2 | Grossman et al., 2014 conference abstract | CSF p-tau181, total tau, p-tau:t-tau ratio | Conference abstract/duplicate of the full peer-reviewed study; no additional extractable domain-specific cognition data. | Excluded |
| 3 | Süssmuth et al., 2003 | CSF tau, S100β, serum creatine kinase | Evaluated disease-stage biomarker changes and ALS-control differences, not cognition-specific outcomes. | Supportive background only |
| 4 | Jiménez-Jiménez et al., 2005 | CSF total tau | ALS-control biomarker comparison; no cognitive assessment or cognition-domain association. | Excluded |
| 5 | Paladino et al., 2009 | CSF total tau | Evaluated tau as a diagnostic/progression biomarker; no cognition-domain-specific analysis. | Excluded |
| 6 | Bourbouli et al., 2017 | CSF TDP-43, Aβ42, total tau, p-tau181 | Diagnostic ALS/FTD biomarker study; did not report direct CSF tau/amyloid associations with ALS cognitive domains. | Supportive background only |
| 7 | Schreiber et al., 2018 | CSF NfL, total tau, p-tau181, p-tau:t-tau ratio | Focused on diagnostic/prognostic value, motor phenotype, MRI, and survival; no ALS cognition-domain-specific CSF tau/amyloid outcome. | Supportive ALS biomarker context |
| 8 | Scarafino et al., 2018 | CSF total tau, p-tau, p-tau:t-tau ratio | Diagnostic and prognostic CSF tau study; cognition-stratified or domain-specific data were not extractable. | Supportive background only |
| 9 | Kasai et al., 2019 | CSF/plasma NfL, TDP-43, total tau | Diagnostic/prognostic biomarker study; not focused on tau/amyloid cognition-domain associations. | Broader ALS biomarker context |
| 10 | Falzone et al., 2022 | Serum NfL, UCHL1, GFAP, total tau | Serum biomarker study; GFAP related to cognitive-behavioral impairment, but no CSF tau/amyloid cognition-domain association. | Broader biomarker discussion |
| 11 | Gong et al., 2022 | CSF p-tau and cognition in ALS | Retracted publication; therefore ineligible for evidence synthesis. | Excluded completely |
| 12 | Nakayama et al., 2013 | CSF total tau, p-tau, Aβ42 | Focused on Kii ALS/parkinsonism-dementia complex and diagnostic differentiation; not a typical ALS cohort with cognition-domain-specific biomarker associations. | Excluded |
| 13 | Mastrangelo et al., 2023 | Plasma GFAP, AD co-pathology, CSF A/T status | Relevant to AD co-pathology in ALS, but primary analysis focused on plasma GFAP and AD co-pathology classification rather than direct CSF tau/amyloid-domain cognition associations. | Supportive discussion only |
| 14 | Thapa et al., 2023 | Tau proteins in ALS | Systematic review/meta-analysis, not an original primary study; diagnostic focus rather than cognition-domain synthesis. | Background only |
| 15 | Agah et al., 2024 | CSF/blood NfL, t-tau, p-tau, Aβ42 | Systematic review/meta-analysis; not an original eligible cohort and not focused on ALS cognition-domain associations. | Background only |
| 16 | Abu-Rumeileh et al., 2025 | Serum and muscle p-tau181/p-tau217 | Blood and muscle biomarker study; not CSF-based and not a CSF tau/amyloid cognition-domain study. | Broader biomarker discussion |
| 17 | Ginanneschi et al., 2025 | Routine CSF, t-tau, p-tau, Aβ42 | Evaluated clinical/prognostic value of lumbar puncture and survival/progression associations; no cognition-domain-specific CSF biomarker analysis. | Supportive background only |
| 18 | Chen et al., 2025 | CSF Aβ, tau, axonal, synaptic, glial, neural, and inflammatory biomarkers | Large CSF biomarker panel focused on ALS diagnostic biomarker discovery; no primary cognition-domain-specific tau/amyloid association. | Broader biomarker discussion |
| 19 | Mukhija et al., 2025 | Multimodal clinical, CSF, serum, and imaging biomarkers | ALS/FTD diagnostic classification study; included tau/amyloid ratios but did not directly test CSF tau/amyloid associations with ALS cognitive domains. | Supportive background only |
| 20 | Kasper et al., 2026 plasma p-tau181 study | Plasma p-tau181 and NfL | Plasma biomarker study, not CSF; therefore outside primary CSF tau/amyloid eligibility criteria. | Broader biomarker discussion |
| 21 | Additional ALS diagnostic CSF biomarker studies | CSF NfL, TDP-43, tau, or mixed panels | Excluded because the primary outcome was ALS diagnostic discrimination rather than cognition-specific biomarker association. | Grouped exclusion |
| 22 | Additional ALS prognostic biomarker studies | CSF or blood neurodegenerative biomarkers | Excluded because outcomes focused on survival, progression, ALSFRS-R, or motor phenotype rather than cognitive domains. | Grouped exclusion |
| 23 | Additional serum/plasma biomarker studies | Blood NfL, GFAP, tau, inflammatory markers | Excluded because biomarkers were not CSF tau/amyloid measures. | Grouped exclusion |
| 24 | Additional AD-only or non-ALS neurodegenerative biomarker studies | CSF tau and amyloid | Excluded because the study population did not include eligible ALS or ALS–FTD-spectrum patients. | Grouped exclusion |
| 25 | Additional imaging/PET/MRI cognition studies | Imaging biomarkers | Excluded because they did not evaluate CSF tau or amyloid biomarkers. | Grouped exclusion |
| 26 | Additional proteomic or inflammatory biomarker studies | Broad CSF/blood protein panels | Excluded because tau/amyloid biomarkers were not the primary analytes or cognition-domain-specific CSF associations were not reported. | Grouped exclusion |
| 27 | Additional review articles | Narrative/systematic reviews | Excluded because they were not original primary studies. | Background only |
| 28 | Additional conference abstracts | Abstract-only reports | Excluded because full peer-reviewed data and extractable cognitive-domain biomarker results were unavailable. | Excluded |
| 29 | Additional case reports/small case series | Variable biomarkers | Excluded because study design did not meet eligibility criteria for observational cohort/case-control synthesis. | Excluded |
| 30 | Additional records with insufficient extractable data | Tau/amyloid or related biomarkers | Excluded because cognition-domain-specific CSF biomarker data could not be extracted. | Grouped exclusion |

**Table note:** This table accounts for the 30 full-text records excluded after eligibility assessment. Individually relevant near-miss studies are listed by study name, while remaining full-text exclusions are grouped by prespecified exclusion category. Studies were excluded from the primary synthesis if they did not report cognition-domain-specific associations between CSF tau and/or amyloid biomarkers and ALS cognitive outcomes.
