# Supplementary material for: CSF Tau and Amyloid Biomarkers in Cognitive Impairment Across the ALS–FTD Spectrum: A Systematic Narrative Review and Evidence Map
Source: Brain Behav. 2026 Sep 1;16(9):e71740. doi: 10.1002/brb3.71740 (PMC13531366; doi:10.1002/brb3.71740)
Supplement: Supplementary file 3 [file BRB3-16-e71740-s003.docx]

## Supplementary Material 1. Search Strategy

A comprehensive literature search was performed in PubMed, Scopus, Web of Science, and Google Scholar from database inception through May 2026. Search terms combined concepts related to amyotrophic lateral sclerosis, cognitive impairment, cerebrospinal fluid biomarkers, tau, and amyloid biomarkers. No language restriction was applied during the initial search. Reference lists of eligible studies and relevant review articles were also manually screened.

### PubMed Search Strategy

("amyotrophic lateral sclerosis"[MeSH Terms] OR "amyotrophic lateral sclerosis"[Title/Abstract] OR ALS[Title/Abstract] OR "motor neuron disease"[Title/Abstract] OR "motor neurone disease"[Title/Abstract])
AND
("frontotemporal dementia"[Title/Abstract] OR FTD[Title/Abstract] OR cognition[Title/Abstract] OR cognitive[Title/Abstract] OR "cognitive impairment"[Title/Abstract] OR neuropsychological[Title/Abstract] OR ECAS[Title/Abstract] OR "Edinburgh Cognitive and Behavioural ALS Screen"[Title/Abstract] OR MoCA[Title/Abstract] OR MMSE[Title/Abstract] OR "ALS Cognitive Behavioral Screen"[Title/Abstract])
AND
("cerebrospinal fluid"[Title/Abstract] OR CSF[Title/Abstract])
AND
(tau[Title/Abstract] OR "total tau"[Title/Abstract] OR t-tau[Title/Abstract] OR "phosphorylated tau"[Title/Abstract] OR p-tau[Title/Abstract] OR p-tau181[Title/Abstract] OR amyloid[Title/Abstract] OR "amyloid beta"[Title/Abstract] OR Aβ42[Title/Abstract] OR Aβ40[Title/Abstract] OR "Aβ42/Aβ40"[Title/Abstract] OR biomarker*[Title/Abstract])

### Scopus Search Strategy

TITLE-ABS-KEY("amyotrophic lateral sclerosis" OR ALS OR "motor neuron disease" OR "motor neurone disease")
AND
TITLE-ABS-KEY("frontotemporal dementia" OR FTD OR cognition OR cognitive OR "cognitive impairment" OR neuropsychological OR ECAS OR "Edinburgh Cognitive and Behavioural ALS Screen" OR MoCA OR MMSE OR "ALS Cognitive Behavioral Screen")
AND
TITLE-ABS-KEY("cerebrospinal fluid" OR CSF)
AND
TITLE-ABS-KEY(tau OR "total tau" OR t-tau OR "phosphorylated tau" OR p-tau OR p-tau181 OR amyloid OR "amyloid beta" OR Aβ42 OR Aβ40 OR "Aβ42/Aβ40" OR biomarker*)

### Web of Science Search Strategy

TS=("amyotrophic lateral sclerosis" OR ALS OR "motor neuron disease" OR "motor neurone disease")
AND
TS=("frontotemporal dementia" OR FTD OR cognition OR cognitive OR "cognitive impairment" OR neuropsychological OR ECAS OR "Edinburgh Cognitive and Behavioural ALS Screen" OR MoCA OR MMSE OR "ALS Cognitive Behavioral Screen")
AND
TS=("cerebrospinal fluid" OR CSF)
AND
TS=(tau OR "total tau" OR t-tau OR "phosphorylated tau" OR p-tau OR p-tau181 OR amyloid OR "amyloid beta" OR Aβ42 OR Aβ40 OR "Aβ42/Aβ40" OR biomarker*)

### Google Scholar Search Strategy

The following keyword combinations were searched in Google Scholar, and the first 200 results sorted by relevance were screened:

"amyotrophic lateral sclerosis" CSF tau cognition
"amyotrophic lateral sclerosis" cerebrospinal fluid amyloid cognition
ALS ECAS tau CSF
ALS cognitive impairment amyloid beta CSF
ALS frontotemporal dementia tau amyloid CSF biomarkers
ALS p-tau181 cognition cerebrospinal fluid
ALS Aβ42 Aβ40 cognition
ALS CSF biomarkers cognitive impairment

### Manual Search

Reference lists of all included studies and relevant review articles were manually screened to identify additional potentially eligible studies. Articles identified through manual searching were assessed using the same eligibility criteria as database-derived records.
